# Thermodynamic bifurcation in anoxic heart: A far-from-equilibrium dissipative structure

**DOI:** 10.1371/journal.pone.0298979

**Published:** 2024-03-07

**Authors:** Yves Lecarpentier, Olivier Schussler, Victor Claes, Jean-Louis Hébert, Xénophon Krokidis, Alexandre Vallée

**Affiliations:** 1 Centre de Recherche Clinique, Grand Hôpital de l’Est Francilien, Meaux, France; 2 Département de Chirurgie Thoracique, Hôpital Cochin, Hôpitaux Universitaires Paris Centre, Paris-Descartes Université, Assistance Publique-Hôpitaux de Paris, Paris, France; 3 Department of Pharmaceutical Sciences, University of Antwerp, Wilrijk, Belgium; 4 Institut de Cardiologie, Hôpital de la Pitié-Salpêtrière, Assistance Publique-Hôpitaux de Paris, Paris, France; 5 Department of Epidemiology and Public Health, Foch Hospital, Suresnes, France; Instituto Nacional de Medicina Genomica, MEXICO

## Abstract

Thermodynamic consequences of a three-hour long anoxia were investigated on the isolated mammalian rat myocardium. The anoxic heart operated in a far-from-equilibrium manner as attested by the non-linearity between the thermodynamic force and the thermodynamic flow. When subjected to slight fluctuations due to anoxia, the open far-from-equilibrium cardiac system presented a thermodynamic bifurcation at ~ 60 minutes of anoxia. The bifurcation was characterized by a sudden change of direction in the bifurcation diagram of a one-dimensional nonlinear differential equation with one parameter and occurred at a non-hyperbolic fixed point at which moment the heart lost its thermodynamic stability. The parameter of the differential equation was the single force of the myosin molecular motor. These results helped to reflect a self-organized process and the occurrence of a dissipative structure. This offers valuable insights into our understanding of myocardial protection and could be of considerable interest, especially for heart transplants where the recipient must benefit from the donor’s heart in the shortest possible time.

## Introduction

Despite a marked decline in cardiovascular deaths over recent decades, coronary insufficiency remains a major cause of cardiovascular morbidity and mortality in industrialized countries. Insufficient myocardial oxygenation leads to cardiac ischemia, the ultimate form of which is myocardial infarction and heart failure [1]. Approximately 3,500 heart transplants are performed each year worldwide and post-operative survival periods average 15 years. In heart transplants, the time between heart removal from the donor and transplantation in the recipient is of critical importance and should not exceed 4–5 hours. Understanding the thermodynamic status of the heart under anoxic stress is thus of the utmost importance.

Based on the concept of self-organization developed by Ilya Prigogine and his colleagues in the 1970s [2–6], amplified slight fluctuations in far-from-equilibrium phenomena can lead to the occurrence of a new orderliness in complex open systems. Prolonged anoxia could profoundly modify the thermodynamic status of the heart. A method was used to identify the occurrence of a bifurcation induced in the myocardium subjected to prolonged anoxia. It established a one-dimensional nonlinear differential equation with one parameter (Ψ) that showed a sudden change of direction on the thermodynamic bifurcation diagram for a given value of Ψ. This differential equation was characterized by a non-hyperbolic fixed point corresponding to the appearance of a thermodynamic bifurcation.

In normal oxygenated conditions, the heart operates in stationary and near-equilibrium, with an affinity less than 2500 Joules and a thermodynamic flow which varies proportional to the thermodynamic force [7]. In our study, prolonged anoxia (3h) induced slight fluctuations on thermodynamic properties of the heart which deviated towards a far-from-equilibrium behavior as assessed by the loss of linearity between the thermodynamic flow and the thermodynamic force. A one-dimensional non-linear differential equation with one parameter (Ψ) was determined. The parameter (Ψ) represented the value for which the bifurcation diagram y = f (Ψ) dramatically changed its direction. This occurred at about 60 min of anoxia. This bifurcation occurred at the non-hyperbolic fixed point of the differential equation, at which level the heart lost it thermodynamic stability. The heart is an open living system and exchanges energy and matter with the exterior. The slight fluctuations due to anoxia, the far-from-equilibrium status induced by anoxia and the reversibility of thermodynamic anomalies of the open system suggested that the anoxic heart was a dissipative structure.

## Materials and methods

### Ethical statement

Experiments conformed to the Guide for Care and Use of Laboratory Animals. The protocol was approved by the Ethical Committee of the Institut National de la Santé et de la Recherche Médicale (INSERM), Paris France. The research complies with the commonly accepted ’3Rs’. No written or verbal consents were requested as the article was focused on rats.

### Experimental protocol

A similar model of anoxia in rat heart was used in a previous study but the protocol was based on tribological properties of biological tissues [8]. In the present study, the thermodynamic consequences applied to anoxic rats was quite different. Twenty rats were sacrificed after anesthesia with intra-peritoneal injection of sodium pentobarbital (60 mg / kg ip). When animals were deeply asleep, the heart was quickly extracted from the thorax. The anterior left ventricular papillary muscle (LVPM) was excised. The LVPM was then quickly mounted in a tissue chamber which contained a Krebs-Henseleit solution (in mmol): 118 NaCl; 24 NaHCO3; 4.7 KCl; 1.2 MgSO4 7H2O; 1.1 KH2PO4; 2.5 CaCl2 6H2O; 4.5 glucose. In control normoxic conditions, the solution was bubbled with 95% O2-5% CO2 to maintain a pH at 7.4. Then, the LVPM was subjected to anoxia (95% N_2_-5% CO_2_) for 3 hours, at a constant temperature (29°C). The LVPM was electrically stimulated by means of two platinum electrodes (electrical stimulus frequency:12/min). Mechanical performance [9] was registered every 30 minutes (0, 30, 60, 90, 120, 150 and 180 min). The electromagnetic lever system has been described earlier [7]. The protocol was carried out at Lo, the initial resting length corresponding to the apex of the Frank-Starling active tension versus initial length curve. Maximum unloaded shortening velocity (Vmax, in Lo. s-1) was measured by means of the zero-load clamp technique [10]. Peak isometric tension of LVPM, i.e. peak force normalized per cross-sectional area (in mN.mm^−2^) was measured from the fully isometric contraction. The hyperbolic tension-velocity (T-V) relationship was constructed by means of 8–10 afterload contractions from zero-load to isometric tension [11] and was fitted according to A.V. Hill’s equation:

(T+a)(V+b)=[To+a]b (1)

where—a and—b were the asymptotes of the Hill hyperbola. For all LVPMs, the T-V relationship was accurately fitted with a hyperbola. The G curvature of the T-V relationship was equal to To / a = Vmax / b [11, 12].

#### A. Huxley formalism

**Fig 1:**
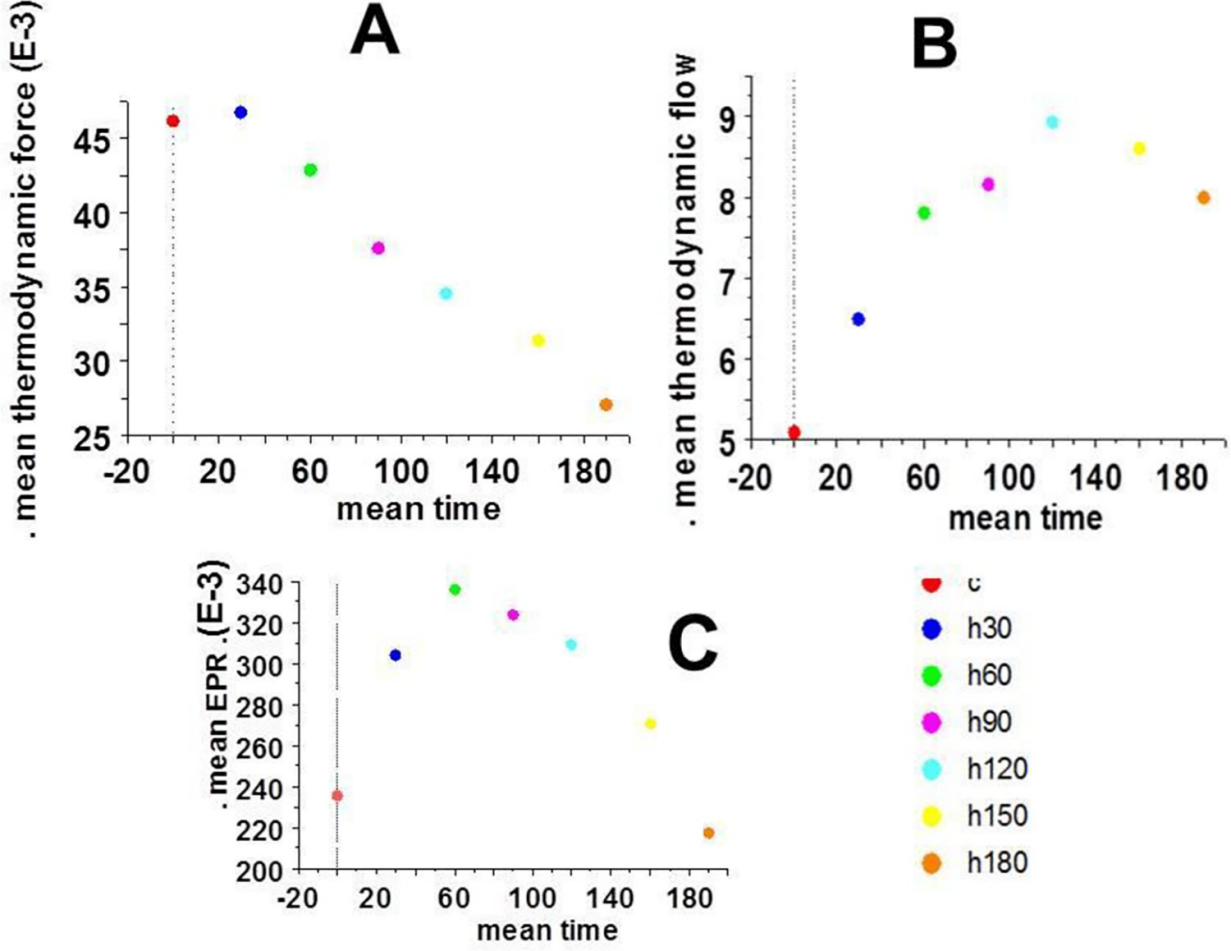
Thermodynamic force, thermodynamic flow ad EPR. Fig 1 represented the mean values of the thermodynamic force (A), of the thermodynamic flow (B), and of the mean value of the entropy production rate (EPR) (C). The mean value of EPR was maximum at 60 min of anoxia. EPR was equal to vo * myosin content / T and thus its derivative according to time was 0 for t = 60 min.

The phenomenological equations developed by A. Huxley [13] allow the calculation of the molecular properties of myosin crossbridges (CBs). To be able to use this formalism, contractile systems must present a hyperbolic T-V relationship, as the asymptotes—a and—b and the curvature G of the T-V relationship were part of the Huxley equations. Using this formalism, the rate of total energy release (E_Hux_) and isotonic tension (P_Hux_) as a function of muscle velocity (V) were obtained by the following equations:

$$EE\;E_{\mathrm{Hux}}=(Ne)(h/2l)(f_{1}/(f_{1}+g_{1}))\{g_{1}+f_{1}(V/\Phi )[(1‐exp(‐\Phi /V)]\}$$ (2)

$$FE\;P_{\mathrm{Hux}}=N(w/l)(f_{1}/(f_{1}+g_{1})\{1‐(V/\Phi )[(1‐exp(‐\Phi /V))(1+(1/2){\left((f_{1}+g_{1})g_{1}/g_{2}\right)}^{2}(V/\Phi ]\}$$ (3)

where f_1_ was the peak value of the rate constant for CB attachment; g_1_ and g_2_ were the peak values of the rate constants for CB detachment; w was the maximum mechanical work of a unitary CB (w / e = 0.75) and e was the free energy required to split one ATP molecule. One ATP was split per CB cycle. The standard free energy ΔG°’_ATP_ was roughly– 60 kJ /mol and the value used for e was 10 ^−19^ J [14]. The tilt of the myosin head relative to actin varied from 0 to h; f_1_ and g_1_ represented a tilt from 0 to h, and g_2_ represented a tilt > h; Φ = (f_1_ + g_1_) h / 2 = b; N was the cycling CB number per mm^2^ at peak isometric tension. The molecular step size h was defined by the translocation distance of the actin filament per ATP hydrolysis produced by the swing of the myosin head. The parameter l was the distance between two successive actin sites with which any myosin site could combine. In accordance with A. Huxley conditions (l >>h), the values of h and l were h = 10 nm and l = 28.6 nm respectively (close to the semi-helicoidally turn of the actin filament) [15]. The estimated value of h was supported by the three-dimensional head structure of the muscle myosin II [16–18]. Calculations of f_1_, g_1_, and g_2_ were made using the following equations:

$$G=f_{1}/g_{1}$$ (4)

$$g_{1}=2wb/ehG$$ (5)

$$g_{2}=2Vmax/h$$ (6)

$$kcat=(h/2l)x[\left(f_{1}g_{1})/(f_{1}+g_{1}\right)]$$ (7)

$$po=(w/l)x[\left(f_{1})/(f_{1}+g_{1}\right)]$$ (8)

where N was the number of active CBs / mm^2^, was the ratio of the peak isometric tension and the unitary CB force (po). The velocity of crossbridge vo = h / ts (ts: time stroke). Myosin content was calculated from the CB number per g of tissue (nM.g^−1^) and the Avogadro number. The myosin ATPase activity was the product of kcat and myosin content. The rate of mechanical work (W_M_) was equal to P_Hux_. V [12]. At any given load level, the efficiency of the contractile tissue was defined as the ratio of W_M_ and E. The maximum value was the max.Efficiency. The thermodynamic force, the thermodynamic flow and the entropy production rate are described in Fig 1.

### Determination of a one-dimensional non-linear differential equation with one parameter Ψ

The rate of entropy production (EPR) was the product of the thermodynamic flow and the thermodynamic force. Let EPR = y = v0 x myosin content / T. This equation can be expressed as a polynomial function of time. The derivative y’ according to time was also a polynomial function. We applied then a polynomial regression between y and y’ and obtained a relationship between y and y’. We found a one-dimentional non-linear differential equation. A term (p0: single CB force) of this equation was related to y’ which was introduced in the differential equation. A one-dimentional non-linear differential equation with one parameter was obtained. We then studied the phase diagram and the bifurcation diagram. On the phase diagram, y’ = f (y, Ψ) shows a non-hyperbolic fixed point (y*) because λ = f ‘(y*) was equal to 0.

### Stability conditions of the thermodynamic cardiac system

The phase diagram at a non-hyperbolic fixed point is structurally unstable. The local stability of the one-dimensional non-linear differential equation y’ = f (y, Ψ) was determined by examining the first derivative of the function y evaluated at y*. The slope of the phase diagram y’ = f (y, Ψ) at the fixed point y* determined the stability of the differential equation.

### Bifurcation diagram

The bifurcation diagram y = f (Ψ) showed a dramatic change of direction at about 60 min of anoxia. This was a bifurcation, occurring at the non-hyperbolic fixed point of the one-dimensional non-linear differential equation of the far-from-equilibrium cardiac system.

## Results

### Mechanical properties of left ventricular papillary muscles (LVPMs) and molecular myosin crossbridge (CB) characteristics

In Tables 1 and 2 are presented the mechanical and biochemical parameters of the LVPMs and CB characteristics.

**Table 1:** Mechanical and biochemical parameters of LVPMs and CB.

Anoxia duration (min)	Vmax (Lo/s)	Tension (mN/mm^2^)	f1 (s^-1^)	g1 (s^-1^)	g2 (s^-1^)
**C vs 180**	***	***	**NS**	***	***
**180 vs r90**	**§§§**	**§**	**NS**	**§§§**	**§§§**
**C vs r90**	**NS**	**!!!**	**NS**	**NS**	**NS**
**0**	3.8±0.7	51±19	314±83	199±69	753±148
**30**	3.6±0.8	44±17	369±102	281±108	726±166
**60**	3.2±0.8	37±14	364±96	321±114	639±160
**90**	2.9 ±0.8	30±14	367±127	357±140	583±168
**120**	2.5±0.8	28±12	359±103	388±118	491±151
**150**	2.2±0.8	22±10	331±85	383±109	444±126
**180**	2.0±0.8	20±8	306±108	358±143	398±141
**r30**	2.9±0.7	32±12	283±96	201±100	581±149
**r60**	3.2±0.7	38±13	276±73	174±60	639±139
**r90**	3.4±0.9	38±15	290±100	157±99	673±174

Mean values ± SD of Vmax, tension, f1, g1 and g2 are shown. Statistics: control values (C) versus 180 min anoxia (*** < 0.001; NS: non significant); 180 min anoxia versus 90 min of oxygen recovery (§ < 0.05; §§§ < 0.001; NS: non significant); C versus 90 min of oxygen recovery (!!! < 0.001; NS: non significant); r30: oxygen recovery at 30min.

**Table 2:** Mechanical and biochemical parameters of LVPMs and CB.

Anoxia duration (min)	Myosin content (n.mol/g)	v0 (mm. s^-1^)	max.Efficiency (%)	CB force (pN)
**C vs 180**	* **	***	***	***
**180 vs r90**	§	§§§	§§§	§§§
**C vs r90**	!	NS	NS	NS
**0**	13.9±5.4	5.1±1.9	28±2	1.6±0.1
**30**	14.1±5.2	6.5±2.5	26±3	1.5±0.1
**60**	12.9±4.8	7.8±3.1	22±2	1.4±0.1
**90**	11.4±4.6	8.2±3.5	21±2	1.3±0.1
**120**	10.5±4.6	8.9±2.8	19±2	1.2±0.1
**150**	9.5±3.6	8.6±3.1	18±2	1.2±0.1
**180**	7.9±3.4	8.0±3.0	18±2	1.2±0.1
**r30**	10.4±3.9	4.6±2.4	27±3	1.5±0.1
**r60**	11.2±3.9	4.2±1.9	28±2	1.6±0.1
**r90**	11.1±4.9	4.1±2.2	28±3	1.6±0.1

Mean values ± SD of myosin content, v0, max.efficiency and CB force are presented. Statistics: control (C) values versus 180 min anoxia (*** < 0.001; NS: non significant); 180 min anoxia versus 90 min of oxygen recovery (§ < 0.05; §§§ < 0.001; NS: non significant); C versus 90 min of oxygen recovery ( ! < 0.05; NS: non significant); r30: oxygen recovery at 30min.

**Fig 2:**
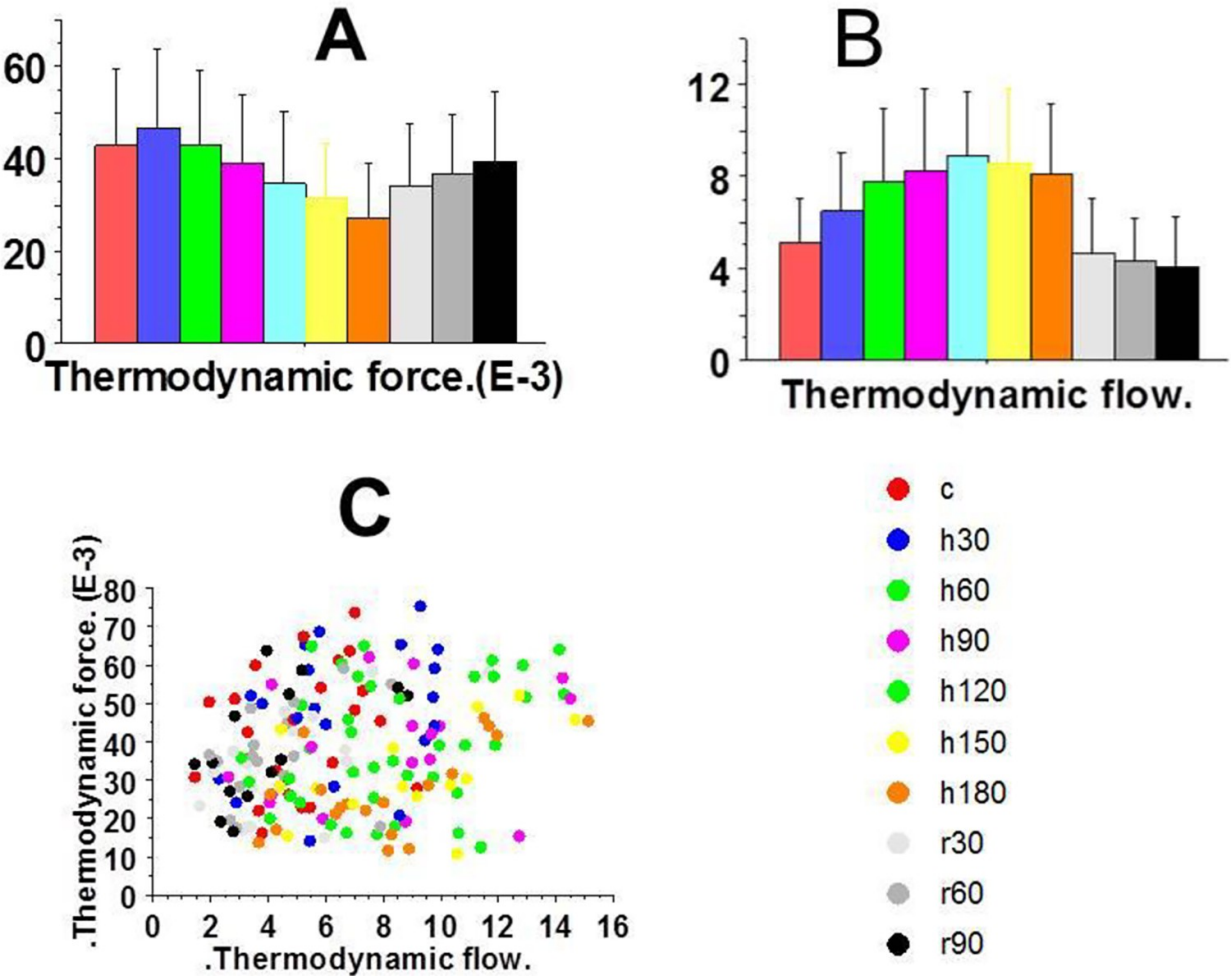
Thermodynamic characteristics of myosin CB. A: Thermodynamic force as a function of time (nmol/g/T); It decreased from t = 30 to t = 180 min; B: Thermodynamic flow as a function of time (mμ/s); it reached a maximum at t = 120 min. C: Relationship between thermodynamic flow and thermodynamic force. The relationship between thermodynamic force and thermodynamic flow indicates the far-from-equilibrium behavior of the cardiac system under prolonged anoxia and the nonlinear link between them.

Thermodynamic force (Fig 2A) and thermodynamic flow (Fig 2B) are presented as a function of time. Thermodynamic flow increased from 0 min to 120 min of anoxia and thereafter decreased from 120 to 180 min of anoxia. Thermodynamic force non significantly increased from zero min to 30 min and thereafter decreased until 180 min. Fig 2C shows the complex non-linear relationship between these two parameters. The non-linear behavior between these two parameters means that the cardiac system operated far-from-equilibrium.

## Determination of a one-dimensional non-linear differential equation with one parameter Ψ

### One-dimensional non-linear differential equation

The entropy production rate (EPR) is the product of the thermodynamic flow and the thermodynamic force. Let y = EPR = vo * myosin content / T. This equation can be expressed as a function of time by a third degree polynomial:

y = 0.237 + (0.003 t)—(2.4 E-5 t^2^) + (4.2 E-8 t^3^); R^2^ = 0.99.

**Fig 3:**
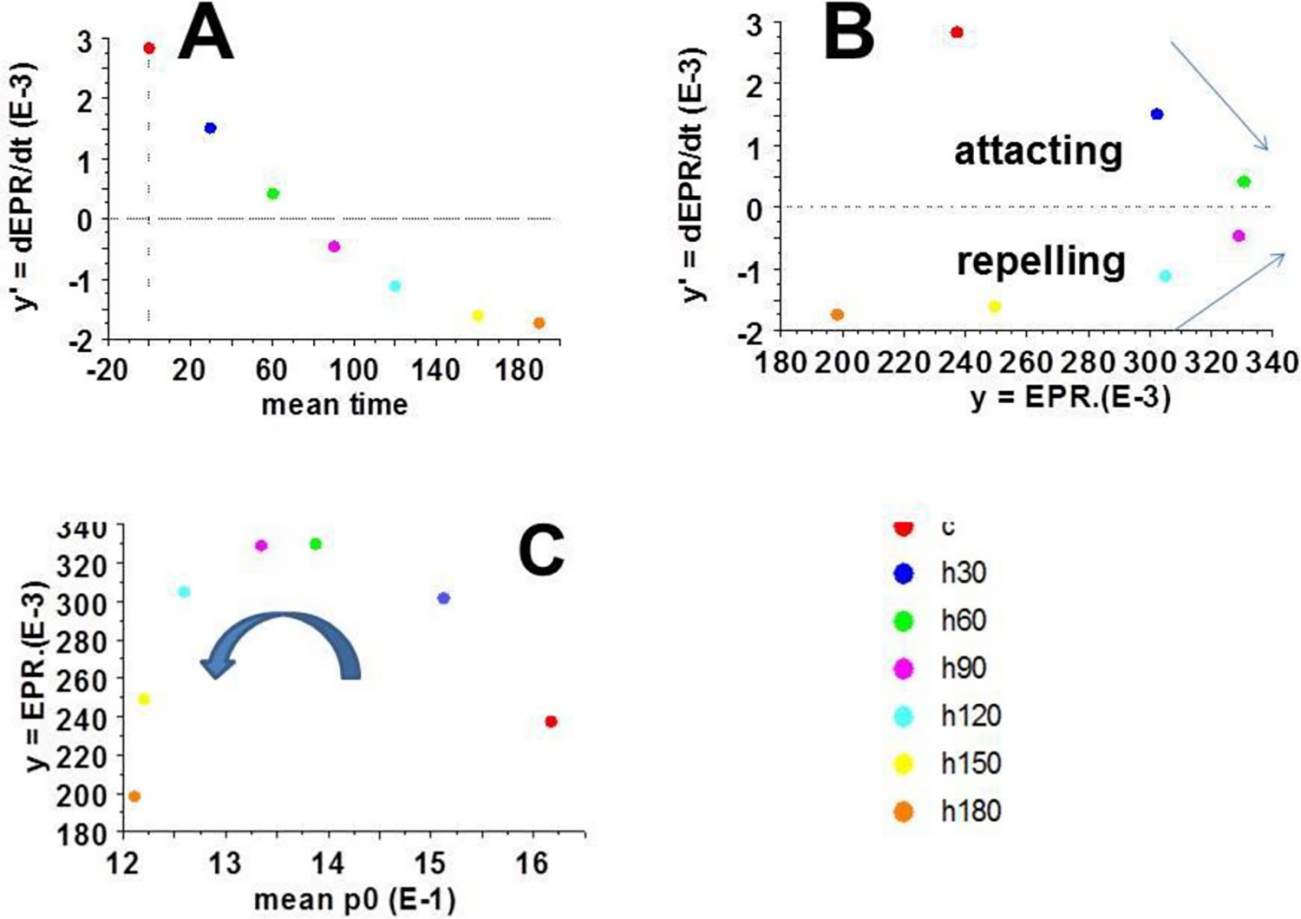
y’ = d(EPR) / dt relationship as a function of time, phase diagram and bifurcation diagram. A: y’ as a function of time; it became negative between 60 and 90 min; B: y’ = d (EFR) / dt as a function of y = EPR; it was attracting for positive values of y’; it was repelling for negative values of y’; C: y = f(Ψ), the bifurcation diagram. Note the change of direction after 60 min.

The derivative y’ according to time is shown in Fig 3A:

y’ = dy / dt = 0.003 - (4.8 E-5 t) + (12.8 E-8 t ^2^); R^2^ = 0.95 (Fig 3A).

Let y = f (y’), a function of y’:

y = 1.359 y’ - 30830 y’^2^ + 6177583 y’^3^; R^2^ = 0.95

This was a one-dimensional non-linear differential equation.

The equation y’ = f(y,Ψ) represented the phase diagram (Fig 3B).

### Determination of the parameter Ψ of the one-dimensional non-linear equation

We showed that y = 1.359 y’– 30830 y’^2^ + 6177583 y’^3^; R^2^ = 0.90.

The term y’ was linearly related to the force of a single CB force (po):

y’ = [(- 20.5 + 15 po) / 1000] / 1.36

Thus, the differential equation was expressed according to the parameter po which was the force of a single CB:

y = ((15po—20.5) / 1000)—(30830 y’^2^) + (6177583 y’^3^); R^2^ = 0.95

We obtained a one-dimensional non-linear differential equation with one parameter po. A bifurcation occurred for po equal to 1.36 pN (Fig 3C), corresponding to 60 min of anoxia.

### Phase diagram: y’ = f(y, Ψ)

The phase diagram y’ = f (y,Ψ) (Fig 3B) shows a non-hyperbolic fixed point (y*) because f ‘(y*) was equal to 0. It occurred at a time about 60 min of anoxia. The phase diagram at a non-hyperbolic fixed point was structurally unstable.

### Bifurcation diagram y = f (Ψ)

The bifurcation diagram y = f (Ψ) presented a dramatic change of direction at ~60 min of anoxia (Fig 3C). When the parameter of the differential equation (Ψ = po) decreased from 1.6 to 1.35, the function y increased. At po = 1.35, there was a sudden change in the y direction. When po continued to decrease, y then suddenly decreased, thus marking the bifurcation. This bifurcation occurred at the non-hyperbolic fixed point of the differential equation of the far-from-equilibrium cardiac system. The value of the parameter Ψ = po was 1.35.

**Stability analysis.** The local stability of the one-dimensional non-linear differential equation y’ = f (y, Ψ) was determined by examining the first derivative of the function y evaluated at y*. Let λ be equal to f ‘(y*). In the present study, λ was equal to 0. Thus y* was a non-hyperbolic fixed point. The slope (positive or negative) of the function y’ = f (y,Ψ) = f(y,po) at the fixed point y* determined the stability of the differential equation. The non-hyperbolic fixed point was half-stable (unstable) because trajectories converged at the fixed point on one side but diverged from the fixed point on the other side. In this hybrid case, the fixed point for duration of anoxia < ~60 min was attracting with a negative slope of y’ = f (y, Ψ). Conversely, the fixed point was repelling with a positive slope of y’ = f (y, Ψ) for duration of anoxia > 60 min.

#### Loss of stability, self-organization and dissipative structures

The heart lost its thermodynamic stability at the fixed point. This was a pre-requisite for self-organization to begin. Dissipative structures were found to appear under the following conditions: 1) the heart must be an open system, which is the case for all living systems; it exchanged energy and matter with its environment; 2) it operated far-from-equilibrium and under a non-linear regime, as attested by the non-linearity between the thermodynamic flow and the thermodynamic force; 3) it was subjected to slight fluctuations during anoxia. This modified the molecular properties of the myosin crossbridges and particularly the attachment and detachment constants. The unitary myosin CB force was depending on them and accounted for the thermodynamic results; 5) these characteristics disappeared as soon as the exchanges with exterior ceased and oxygen recovery induced an almost reversibility of the thermodynamic properties.

#### O2 recovery

Re-oxygenation induced an almost complete reversibility of thermodynamic modifications in anoxic left ventricular papillary muscles. Reversibility was complete for several indices during O2 recovery (Tables 1 and 2). This was the case for maximum unloaded shortening velocity, catalytic rate constants of attachment and detachment, and velocity of sarcomere which was the thermodynamic flow. However, some indices presented a partial reversibility, as was the case for the myosin concentration of tissue which was the thermodynamic force.

## Discussion

Earlier studies have reported systems with self-organization and dissipative structures, such as Turing structures, chemical oscillations, Belousov-Zhabotinsky reactions, Brusselator and Oregonator models, turbulent liquid motion, Bénard cells, and biomolecular asymmetry [6]. In biological complex systems, the occurrence of self-organization is ubiquitous in natural systems. In medicine, the description of self-organization and dissipative structures is not frequent.

We found that the thermodynamic properties of the rat myocardium after prolonged anoxia became deeply modified as it moved far away from equilibrium. Heart muscle as a dynamic biological system operated far-from-equilibrium and presented a thermodynamic bifurcation [3, 6]. This reflected the fact that a phenomenon described by the bifurcation diagram y = f (Ψ) changed its trajectory or its behavior at a certain value of the parameter Ψ = p0 [19]. This was attested by the fact that the thermodynamic branch suddenly changed its behavior once a certain value of the parameter p0 was reached on the bifurcation diagram (1.35pN). The open cardiac system then operated on a new thermodynamic branch. Fixed points and bifurcations characterized the one-dimensional non-linear systems. Limit cycles and biological oscillations are encountered in two-dimensional non-linear systems and chaos and fractals in tri-dimensional non-linear systems. Anoxia induced slight fluctuations on myosin crossbridges whose values of the rate constants for CB attachment and detachment were disturbed. The CB single force depended on them and was equal to po = (w/ l) x [(f_1_) / (f_1_+g_1_)] (Eq 8). The formula of entropy production rate is very close to that of the myosin ATPase activity which is the product of the myosin content and the crossbridge velocity (myosin ATPase activity = MC * v0). This is also the product of MC and the catalytic constant kcat (MC * kcat). Thus, this product becomes negative for a certain value of the parameter Ψ, which reflects the occurrence of a self-organization process and raises the possibility of a dissipative structure, under reserve of supplementary properties (open far-from-equilibrium system exchanging energy and matter with the exterior, subjection to slight fluctuations, and reversibility). This technique can be extended to the thermodynamic analysis of any enzymatic activity.

After the restitution of oxygen, the thermodynamic modifications that had occurred were found to be largely reversible, demonstrating that the deleterious effects induced by anoxia were not definitive. After 3 hours of anoxia, oxygen recovery induced a return in the thermodynamic branch, taking a position very close to that of the initial branch. The appearance of self-organization is ubiquitous in natural systems, particularly in biological complex open systems [20, 21]. The fact that the myocardium reacted in this way to such prolonged anoxia bears witness to the incredible ability of nature to generate a self-organization process capable of resisting and delaying cardiac cell death. The anoxia-induced bifurcation probably prevented the death of a significant number of myosin heads, which would have otherwise impaired the contractile function of the heart. More specifically, the transition to a new thermodynamic branch appears to have prevented, or at least limited, the appearance of any irreversible damage to the myosin CBs.

## Conclusion

Anoxia induced major thermodynamic abnormalities in mammalian heart muscle. Under anoxia, the cardiac system operated in a far-from-equilibrium manner as attested by the non-linearity between the thermodynamic force and the thermodynamic flow. The cardiac system was open, exchanging energy and matter with the exterior, and was subjected to slight fluctuations during anoxia. It returned almost to the control values after oxygen restitution. A bifurcation appeared when a sudden change of direction in the bifurcation diagram of the one-dimensional non-linear differential equation with the parameter Ψ occurred. The bifurcation occurred at a non-hyperbolic fixed point at ~ 60 minutes of anoxia, at which level the heart lost it thermodynamic stability. This was a pre-requisite for self-organization to begin. Along with other characteristics, this testified to the occurrence of a dissipative structure. These observations could be of the utmost importance, especially in the context of heart transplants where the recipient must benefit from the donor’s heart in the shortest possible time.

## References

[1] Wilmot KA, O’Flaherty M, Capewell S, Ford ES, Vaccarino V. Coronary Heart Disease Mortality Declines in the United States From 1979 Through 2011: Evidence for Stagnation in Young Adults, Especially Women. Circulation. 2015;132(11):997–1002. doi: 10.1161/CIRCULATIONAHA.115.015293 26302759 PMC4828724

[2] Prigogine I. Introduction to thermodynamics of Irreversible Processes. New York: Wiley, J; 1967.

[3] Glansdorff P, Prigogine I. Thermodynamics of structure stability and fluctuations. Wiley, J. ed. New York 1971.

[4] Prigogine I, Nicolis G, Babloyantz A. Nonequilibrium problems in biological phenomena. Ann N Y Acad Sci. 1974;231(1):99–105. doi: 10.1111/j.1749-6632.1974.tb20557.x 4522899

[5] Nicolis G, Prigogine I. Self-organization in non-equilibrium systems:From dissipative structures to order through fluctuations. In: Son JWa, editor. <ew York, NY USA1977.

[6] Kondepudi D, Prigogine I. Modern thermodynamics from heat engines to dissipative structures. New York: Wiley & Sons; 1999. 1–486 p.

[7] Lecarpentier Y, Claes V, Krokidis X, A Vallée A. Comparative Statistical Mechanics of Muscle and Non-Muscle Contractile Systems: Stationary States of Near-Equilibrium Systems in A Linear Regime. Entropy Journal 2017;19(10):558.

[8] Lecarpentier Y, Claes V, Hebert JL, Krokidis X, Schussler O, Vallee A. Friction in Myocardial Anoxia Leads to Negative Excess Entropy Production, Self-Organization, and Dissipative Structures. International journal of molecular sciences. 2022;23(13). doi: 10.3390/ijms23136967 35805967 PMC9266918

[9] Brutsaert DL, Claes VA, Goethals MA. Effect of calcium on force-velocity-length relations of heart muscle of the cat. Circulation research. 1973;32(3):385–92. doi: 10.1161/01.res.32.3.385 4691344

[10] Brutsaert DL, Claes VA, Sonnenblick EH. Velocity of shortening of unloaded heart muscle and the length-tension relation. Circulation research. 1971;29(1):63–75. doi: 10.1161/01.res.29.1.63 5561409

[11] Hill AV. The heat of shortening and the dynamic constants of muscle. Proc R Soc Lond Biol Sci. 1938;126:136–95.

[12] Lecarpentier Y, Chemla D, Blanc FX, Pourny JC, Joseph T, Riou B, et al. Mechanics, energetics, and crossbridge kinetics of rabbit diaphragm during congestive heart failure. Faseb J. 1998;12(11):981–9. doi: 10.1096/fasebj.12.11.981 9707170

[13] Huxley AF. Muscle structure and theories of contraction. Prog Biophys Biophys Chem. 1957;7:255–318. 13485191

[14] Veech RL, Lawson JW, Cornell NW, Krebs HA. Cytosolic phosphorylation potential. The Journal of biological chemistry. 1979;254(14):6538–47. 36399

[15] Sheterline P., Clayton J, Sparrow J.C. Protein Profile. 3rd edition ed. London: Academic Press; 1996.

[16] Huxley AF, Simmons RM. Mechanical properties of the cross-bridges of frog striated muscle. J Physiol. 1971;218(1):59P–60P. 5130636

[17] Rayment I, Holden HM, Whittaker M, Yohn CB, Lorenz M, Holmes KC, et al. Structure of the actin-myosin complex and its implications for muscle contraction. Science. 1993;261(5117):58–65. doi: 10.1126/science.8316858 8316858

[18] Dominguez R, Freyzon Y, Trybus KM, Cohen C. Crystal structure of a vertebrate smooth muscle myosin motor domain and its complex with the essential light chain: visualization of the pre-power stroke state. Cell. 1998;94(5):559–71. doi: 10.1016/s0092-8674(00)81598-6 9741621

[19] Strogatz SH, editor. Nonlinear dynmics and chaos. Cambridge: Perseus Books Publishing, LLC 1994.

[20] Camazine S, Deneubourg JL, Franks NR, Sneyd J, Theraulaz G, Bonabeau E. Self-organization in biological systems. In: Press PU, editor. StatPearls. Treasure Island (FL) 2001. p. 1–538.

[21] Goldbeter A. Biological oscillations and cellular rhythms. In: Press CU, editor.1996. p. 1–605.

